# A Contributing Role for Anti-Neuraminidase Antibodies on Immunity to Pandemic H1N1 2009 Influenza A Virus

**DOI:** 10.1371/journal.pone.0026335

**Published:** 2011-10-24

**Authors:** Glendie Marcelin, Rebecca DuBois, Adam Rubrum, Charles J. Russell, Janet E. McElhaney, Richard J. Webby

**Affiliations:** 1 Department of Infectious Diseases, Division of Virology, St. Jude Children's Research Hospital, Memphis, Tennessee, United States of America; 2 Department of Structural Biology, St. Jude Children's Research Hospital, Memphis, Tennessee, United States of America; 3 Center for Immunotherapy of Cancer and Infectious Diseases, Department of Immunology, University of Connecticut School of Medicine, Farmington, Connecticut, United States of America; University of Texas Medical Branch, United States of America

## Abstract

**Background:**

Exposure to contemporary seasonal influenza A viruses affords partial immunity to pandemic H1N1 2009 influenza A virus (pH1N1) infection. The impact of antibodies to the neuraminidase (NA) of seasonal influenza A viruses to cross-immunity against pH1N1 infection is unknown.

**Methods and Results:**

Antibodies to the NA of different seasonal H1N1 influenza strains were tested for cross-reactivity against A/California/04/09 (pH1N1). A panel of reverse genetic (rg) recombinant viruses was generated containing 7 genes of the H1N1 influenza strain A/Puerto Rico/08/34 (PR8) and the NA gene of either the pandemic H1N1 2009 strain (pH1N1) or one of the following contemporary seasonal H1N1 strains: A/Solomon/03/06 (rg Solomon) or A/Brisbane/59/07 (rg Brisbane). Convalescent sera collected from mice infected with recombinant viruses were measured for cross-reactive antibodies to pH1N1 via Hemagglutinin Inhibition (HI) or Enzyme-Linked Immunosorbent Assay (ELISA). The ectodomain of a recombinant NA protein from the pH1N1 strain (pNA-ecto) was expressed, purified and used in ELISA to measure cross-reactive antibodies. Analysis of sera from elderly humans immunized with trivalent split-inactivated influenza (TIV) seasonal vaccines prior to 2009 revealed considerable cross-reactivity to pNA-ecto. High titers of cross-reactive antibodies were detected in mice inoculated with either rg Solomon or rg Brisbane. Convalescent sera from mice inoculated with recombinant viruses were used to immunize naïve recipient Balb/c mice by passive transfer prior to challenge with pH1N1. Mice receiving rg California sera were better protected than animals receiving rg Solomon or rg Brisbane sera.

**Conclusions:**

The NA of contemporary seasonal H1N1 influenza strains induces a cross-reactive antibody response to pH1N1 that correlates with reduced lethality from pH1N1 challenge, albeit less efficiently than anti-pH1N1 NA antibodies. These findings demonstrate that seasonal NA antibodies contribute to but are not sufficient for cross-reactive immunity to pH1N1.

## Introduction

The development of an influenza vaccine that confers broad-spectrum immunity is of paramount importance in the fight against future or re-emerging influenza pandemics. In an effort to discover this vaccine panacea, a myriad of formulations have been developed and pre-clinically tested with varied results [1]–[7]. The continuous antigenic drift of circulating viruses poses a serious challenge towards achieving cross-protection against divergent influenza strains [8], [9]. The influenza vaccine formulations are updated every year to protect against various influenza strains. This severely limits the immunity to future (re-)emerging influenza viruses. Although licensed influenza vaccines are restricted to inducing primarily homotypic protection, studies indicate that natural exposure to seasonal H1N1 influenza A strains induce cross-reactive serum antibodies to the antigenically distinct pandemic H1N1 2009 influenza A virus (pH1N1) [10], [11].

The presence of serum neutralizing antibody responses to the hemagglutinin (HA) protein is a well-established hallmark correlate of protection against influenza infection. Exposure from previous infections or immunization with influenza vaccines from either the 1976 or contemporary 2006–2009 seasons induce cross-reactive neutralizing antibodies to the HA of pH1N1 (pHA) in elderly recipients [10], [12], [13]. The implication is that these neutralizing antibodies may to some degree provide protection against pH1N1 in certain populations. Interestingly, serological analysis of animals previously exposed to contemporary seasonal influenza A/Brisbane/59/07 H1N1 strain show little or no seroconversion to pH1N1 when measured by either hemagglutination inhibition (HI) or microneutralization (MN) based methods [4], [14]. In spite of limited or lack of detectable cross-reactive neutralizing antibodies, prior infection with seasonal strains partially reduces weight loss, virus replication and transmission of pH1N1 in mouse, ferret, guinea pig or porcine challenge models [4], [14]–[16] or limits morbidity in humans [17]. However, only upon multiple infections with contemporary seasonal strains do the severity of infection and level of virus replication greatly decrease after pH1N1 challenge [16]. Taken together, these studies illustrate that repeated exposures to seasonal influenza viruses throughout the life span of humans may be beneficial in not preventing but rather limiting pH1N1-related morbidity. The mere fact that cross-reactive neutralizing antibodies against pHA were not detected in previous studies [4], [14]–[16] and due to the highly variable nature of HA antigenicity warrants investigation into whether another viral moiety of seasonal influenza strains contributes to this cross-protective response.

Several studies point to the importance of the second most abundant surface influenza glycoprotein neuraminidase (NA) in conferring cross-reactive immunity [1], [18], [19]. Case in point, anti-N2 serum antibodies provide protection against antigenically distinct viruses belonging to the same subtype [19]. Also, anti-N1 (H1N1) partially protects mice against H5N1 challenge [18]. The importance of NA in providing immunity against pH1N1 has not yet been defined. Nonetheless, we along with others previously showed that seasonal influenza viruses from 2004–09 seasons possess the capacity to boost cross-reactive serum antibodies to the NA of pH1N1 mainly in elderly individuals [20], [21]. Therefore, it is probable that seasonal NA proteins, which belong to a different genetic lineage than pH1N1 NA protein, can provide cross-protective immune responses.

Little is known regarding the role of cross-reactive antibodies raised against the NA of antigenically distinct seasonal influenza strains [20] on the protective response to pH1N1. Our aim in this report is to evaluate the magnitude of the cross-reactive immune response to the NA of pH1N1 after exposure to the NA of contemporary seasonal H1N1 influenza viruses. Also, do these antibodies to antigenically distinct NA proteins add to immunity against pH1N1? In the context of an influenza infection, we assessed the contributing role of anti-NA antibodies on immunity to pH1N1 using recombinant viruses encoding only one gene (NA) belonging to contemporary seasonal H1N1strains or less recent H3N2. Because ELISA are more sensitive than neuraminidase inhibition assays [22] for detecting NA-specific serum antibodies [23], we used this method in our studies. By ELISA, we show that a single infection with recombinant viruses with a seasonal N1 but not N2 NA gene stimulate high levels of cross-reactive serum antibodies to baculovirus-expressed pNA-ecto from the A/California/04/09 (pH1N1) strain. Furthermore, N1 not N2 NA antibodies contribute partial protection against lethality from wild-type pH1N1 infection. As predicted, a recombinant virus encoding the NA of pH1N1 afforded the greatest degree of immunity against pH1N1 challenge. The results presented here highlight the notion that in addition to HA, the NA component of seasonal influenza vaccines should be optimized in future preparations.

## Methods

### Ethics statement

All experimental procedures conducted in mice were reviewed and approved by St. Jude Children's Research Hospital (SJCRH) Institutional Animal Care and Use Committee, approval number 428. All human study protocols were approved by the University of British Columbia and the Institutional Review Board of the University of Connecticut Health Center. The approval numbers for all human studies are H06-00231, H06-03969, H07-02008, H08-01775, and 03-340.

### Mice

Six-eight week old wild-type (wt) Balb/c or Severe combined immunodeficient, (SCID) mice lacking T and B lymphocytes on the Balb/c background were obtained from Jackson laboratories (Bar Harbor, Maine). All mice were housed in the Animal Resource Center SPF Bio-safety level 2 or 2+ facility at SJCRH.

### Virus propagation

The reverse genetic (rg) 7+1 recombinant viruses with 7 genes from A/Puerto Rico/08/34 (PR8) and the NA gene segment from either A/Solomon/03/06 (rg Solomon), A/Brisbane/59/07 (rg Brisbane), A/California/04/09 (rg California) H1N1 viruses or the A/Aichi/02/68 (rg X−31) H3N2 strain were generated using the 8-plasmid reverse genetic method [24]. The wt H1N1 human influenza viruses, A/New Jersey/11/76 (wt New Jersey), A/Solomon Islands/03/06 (wt Solomon), A/Brisbane/59/2007 (wt Brisbane) or pandemic: A/Tennessee/1-560/2009 (wt Tennessee), A/California/04/09 (wt California) were all obtained from the World Health Organization influenza collaborating laboratories. The recombinant and wt viruses were propagated in the allantoic cavities of 10-day old embryonic chicken eggs. The infection titers of all viruses were determined by using the Reed and Muench method [25], and expressed as the log_10_ of the 50% infectious dose (EID_50_) of fluid.

### Virus sequencing

To confirm that the recombinant viruses did not acquire mutations after virus rescue, RNA was isolated from each virus using the RNeasy minikit (Qiagen, Valencia, CA). RT-PCR was performed using universal NA primers [24]. Sequencing reactions were performed and analyzed using the Lasergene sequencing analysis software (DNASTAR). Only the seasonal H1N1 rg Solomon virus acquired one single amino acid substitution at position 469 changing from threonine to alanine. This substitution lies outside of the major antigenic (immunogenic) regions of NA [26].

### Neuraminidase protein production

Purified NA protein from pH1N1 was prepared as described previously [26], [27]. Synthetic, codon-optimized cDNA encoding an N-terminal 6×His-tag, a 15-residue tetramerization sequence, a thrombin cleavage site, and NA residues 83–468 (in N2 numbering) of the A/California/04/2009 H1N1 influenza virus were cloned into the pAcGP67B plasmid (BD Biosciences, Franklin Lakes, NJ). These residues consisted of the NA ectodomain excluding the cytoplasmic and trans-membrane domains. The ectodomain of NA rather than the full-length protein was synthesized due to the presence of antibody/antigenic sites located on the ectodomain region which also is the only region exposed on the surface of the virion. A baculovirus expression system (Invitrogen, Life Technologies, Carlsbad, CA) was used to express recombinant NA protein ectodomains in Sf9 insect cells. The pNA-ecto protein secreted into the cell culture medium was recovered by metal affinity chromatography using a HisTrap column (GE Healthcare Life Sciences). The pNA-ecto was further purified by size-exclusion chromatography on a Superdex200 16/60 column (GE Healthcare) in 20 mM sodium phosphate pH 8.0 and 150 mM Na Cl. Purified tetrameric pNA-ecto was concentrated to 0.6 mg/ml.

### Immunization of mice

Balb/c mice (n = 20 per group) were inoculated intranasally with either phosphate-buffered saline (PBS) or 10^3^ EID_50_ of rg Solomon, rg Brisbane, or rg California viruses. Because rg X−31 virus is lethal when administered at a dose of 10^3^ EID_50_ mice were infected at a dose of 10^2^ EID_50_.To mimic multiple exposures in humans, Balb/c mice (n = 7 per group) were primed with 10^3^ EID_50_ of either wt New Jersey, wt Brisbane, wt Solomon or wt Tennessee wild-type viruses. Three weeks later, mice were boosted with 10^3^ EID_50_ of wt Solomon or PBS (mice primed with Tennessee). All viruses were diluted in a final volume of 30 µl of PBS. Mice were infected after sedation with isoflurane.

### Sera collection

Sera were collected from each Balb/c mouse via the retro-orbital method three weeks after prime and boost with wt viruses or inoculation with recombinant viruses. In separate studies, SCID mice were bled 1, 4, 7 and 10 days post sera transfer (n = 16 per sera group). Sera samples were collected from each treatment group, heat inactivated for 30 minutes at 56°C and tested for virus-specific antibodies prior to use in passive transfer studies. We received human sera from a prospective study of adults 65–93 years old (median, 74 years), recruited in the Greater Vancouver Area of British Columbia, Canada or in the vicinity of the Greater Hartford Area of Connecticut during the 2007–08 and 2008–09 influenza seasons. A written informed consent was obtained from all participants in the study. All participants received the standard dose of the licensed trivalent split-inactivated (TIV) seasonal influenza vaccine containing A/Solomon Islands/03/2006-like (H1N1), A/Wisconsin/67/2005-like (H3N2), and B/Malaysia/2506/2004-like viruses in 2007–2008 or A/Brisbane/59/2007 (H1N1)-like, A/Brisbane/10/2007 (H3N2)-like, and B/Florida/04/2006-like viruses in 2008–2009 season. Sera were collected from each participant 4 weeks after vaccination.

### Passive transfer of serum antibodies and virus challenge

To assess the cross-protective efficacy of serum antibodies to the NA of seasonal strains, a volume of 100–150 µl of pooled heat inactivated sera collected three weeks after inoculation with recombinant or wt viruses were passively transferred intraperitoneally into naïve recipient Balb/c or SCID mice (n = 5−10 per treatment group). Because each pooled sera could not be normalized to a standard titer of NA antibody without non-specifically diluting HA antibodies, undiluted sera with equivalent HI titers to PR8 (1,280–2,560) were used in each passive transfer study. Recipient animals were intranasally challenged 18–24 hours later with 30 µl of 10^6^ EID_50_ wt California. Animals were monitored daily for mortality and weight loss up to 12 days post-infection (dpi).

### Serum analysis

For HI assays, sera were treated with receptor destroying enzyme (Denka Seiken Co., Ltd., Tokyo, Japan) overnight then tested for HI titers [28] against wt Solomon, wt Brisbane, wt Tennessee, wt California or rg Solomon, rg Brisbane, or rg California. Samples without detectable HI titers were assigned a titer of<40. Antigen-specific Ig (IgA, IgG, and IgM) antibodies in either mouse or human sera were measured by ELISA. Briefly, 96-well microtiter plates (Corning, Lowell, MA) were coated over night at 4°C with purified pNA-ecto. After blocking with 3% dry milk in 1×PBS−0.05% Tween−20 at 37°C, serial dilutions of sera were incubated at room temperature for 1 hour. Antibodies were detected using goat-anti mouse or anti-human Ig conjugated to alkaline phosphatase (Rockland, Immunochemicals Gilbertsville, PA) and *p*-nitrophenyl phosphate as the substrate (KPL Inc., Gaithersburg, MD). The absorbance for each well was measured at 405 nm using a microplate reader (Bio-Rad, Los Angeles, CA). Endpoint antibody titers were defined as the reciprocal of highest sera dilution resulting in an absorbance of ≥0.150. Cytokines were measured in sera collected from mice infected with recombinant viruses. The picogram per milliliter (pg/ml) concentration of 32 mouse cytokines was determined using the multiplex bead system according to the manufacturer instructions (Millipore Corporation, Billercia, MA). Multiplex beads were measured using the Millipore Luminex 200 instrument (Luminex Corporation, Austin, TX) and quantified with the Luminex×Ponent version 3.1 instrumentation software (Luminex Corporation).

### Neuraminidase activity of recombinant viruses or purified pNA-ecto protein

Comparisons of the NA activity in each recombinant virus prep or purified pNA-ecto was tested by using a miniaturized format of the neuraminidase assay [22]. Briefly, using a 96-well polypropylene PCR plate (RPI, Mount Prospect, IL), serial dilutions of either virus diluted in PBS or pNA-ecto diluted in PBS (containing 1–10 mM CaCl_2_ for optimal activity) was incubated with the substrate fetuin (25 mg/ml) overnight at 37°C. The NA activity of each virus preparation or pNA-ecto was determining by calculating the percent increase in the cleavage of fetuin (Optical Density: 550 nm) when compared to PBS only wells.

### Titrations of virus in lungs

Lungs from Balb/c mice were harvested at 5 dpi (n = 5 per treatment group) immediately after mice were euthanized with Avertin (2, 2, 2-tribromoethanol; Sigma Aldrich, St. Louis. MO). Tissue samples were homogenized and processed as described [28]. The titer of virus in each sample was calculated by the Reed and Muench method [25] and expressed as 50% tissue culture infective dose (TCID_50_).

### Statistical analysis and amino acid sequence alignment

For statistical analysis, significant differences in Kaplan-Meier survival curves were determined by using the log rank test (Graph Pad Prism version 5.03). The database GenBank was used to obtain NA amino acid sequences of seasonal (Solomon and Brisbane) and pandemic (Tennessee and California) influenza strains. Because several C-termini residues of both Solomon and Tennessee were not available in GeneBank at the time of the analysis, we retrieved sequences of only the ectodomain for each virus strain. To determine the percent homology between each seasonal and pandemic ectodomain sequences, a clustalW amino acid sequence alignment was performed as described previously [29] (BioEdit Sequence alignment editor version 7.0.9 for, Windows 95/98/NT). Significant differences in the Ig ELISA titers were determined by student's t-test (Excel for Windows XP). Differences in NA activity from recombinant viruses or differences in virus titers in the lung were calculated by the analysis of variance method (ANOVA) followed by post hoc comparison tests (Graph Pad Prism).

## Results

### Seasonal and pandemic 2009 H1N1 influenza strains share high sequence homology in their neuraminidase protein

**Figure 1:**
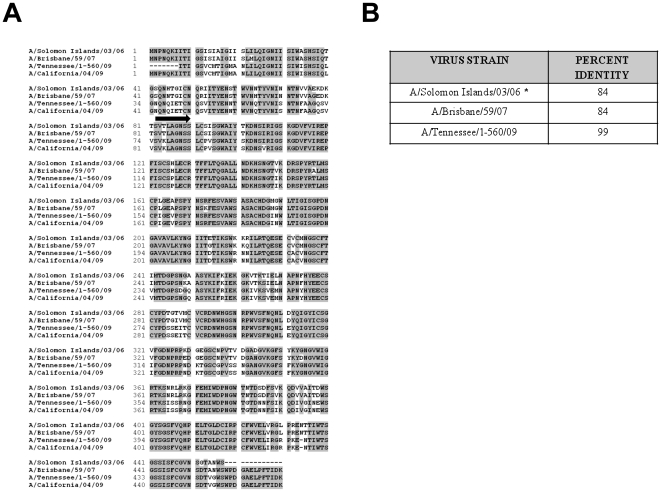
Alignment of amino acid sequences of the neuraminidase from seasonal or pandemic H1N1 influenza viruses. The GenBank sequences of the NA of Solomon, Brisbane, Tennessee and California were aligned using the clustalW alignment program (BioEdit software, Windows 95/98/NT). Numbers to the left of each line indicates amino acid position. Gaps in the alignment are represented as dashed lines. A) Sequences that are shaded in grey indicate identical amino acids between the full length NA of H1N1 viruses. The black arrow indicates the starting sequences of the ectodomain for each virus strain (residue 82 to end).* assumption that the NA sequence of Solomon has identical residues at C-terminus. B) The percent identity of the NA ectodomain between Solomon, Brisbane, Tennessee viruses compared to California was calculated using BioEdit.

Despite the genetic divergence between seasonal influenza viruses versus the recently emerged pH1N1, they share several conserved epitopes [30], [31]. To determine the overall similarity between the NA of these viruses, a sequence alignment at the amino-acid level was performed comparing the NA ectodomain sequence of influenza H1N1viruses. The ectodomain NA of seasonal: Solomon, Brisbane or pandemic: Tennessee viruses were all compared to the NA of pH1N1 California virus. Identical residues are indicated in shaded boxes (Fig. 1A). As predicted, the pandemic strain Tennessee shares the highest identity (99%) with California (Fig. 1B). Contemporary seasonal strains Solomon and Brisbane also share identity to California (84%). These data indicates high percent identity exists within the region containing all of the antigenic epitopes. This supports the idea that antibodies raised against seasonal NA as a result of previous infections or vaccination may cross-react with the NA of pH1N1.

### Characterization of purified NA ectodomain protein from the A/California/04/09 influenza strain

We generated recombinant NA ectodomain from the pH1N1 California strain (pNA-ecto) using a baculovirus insect cell expression system. The purified pNA-ecto was subjected to size exclusion chromatography and found to elute as a single peak at ∼200 kDa, consistent with pNA-ecto forming tetramers (Fig. S1A). The purity of recombinant pNA-ecto was confirmed using denaturing Coomassie blue-staining analysis (Fig. S1B). These data suggest that the pNA-ecto protein is correctly-folded in a native tetrameric form as found on the surface of influenza virions.

Our previous report demonstrates that immunization with TIV boosted the level of cross-reactive serum antibodies to whole-inactivated wt pH1N1 in elderly humans [20]. In the current report, we tested the activity and immunogenicity of purified pNA-ecto. First we tested NA activity using a miniaturized format of the NA assay [22]. Compared to PBS only wells, purified pNA-ecto displayed increased activity only in the presence of calcium chloride [(CaCl_2_) (Fig. S2A)]. Next we used an ELISA-based approach to measure the reactivity between pNA-ecto and sera collected from elderly humans immunized with TIV containing either Solomon or Brisbane H1N1 influenza components. We observed a large cross-reactive response to pNA-ecto after immunization with TIV containing either seasonal H1N1 component (Fig. S2B). There was no significant difference in titer between vaccinees (*P*≥0.27). As predicted, there was no reactivity in pooled sera collected from Balb/c mice inoculated with PBS but a high degree of recognition by pooled convalescent sera from mice infected with wt California (positive control sera, Table 1). In separate studies, convalescent sera from DBA/2 mice infected with the unrelated H3N2 influenza strain, A/Shorebird/Delaware/79/99, displayed minimal cross-reactive titers to pNA-ecto (data not shown), thus demonstrating the specificity of the response to pNA-ecto. These results indicate that the antigenicity, specificity and presumably the structural conformity of pNA-ecto were retained relative to the native form.

**Table 1:** Serum antibody titers in Balb/c mice after priming with reverse genetic 7+1 NA recombinant viruses as determined by hemagglutination inhibition and ELISA assays.

GROUP a	HI	HI	ELISA (Ig) b
7+1 rg viruses c	Homologous HA d	California HA e	California NA f
NA Solomon	1,280	<40	640
NA Brisbane	2,560	<40	320
NA California	1,280	<40	10,240
NA X−31	2,560	<40	80
PBS g	<40	<40	40
Positive Control h	<40	640	102,400

a Each group of mice primed intranasally with 7+1 recombinant viruses.

b ELISA titers expressed as reciprocal of the highest dilution of pooled sera with an O.D. value of≥0.150 when tested against purified NA ectodomain of A/California/04/09.

c Recombinant viruses with 7 gene segments from PR8 and the NA segment from the indicated H1N1 or H3N2 influenza A strain.

d Pooled sera tested for HI titers against rg PR8.

e Pooled sera tested for HI titers against wild-type A/California/04/09.

f Pooled sera tested for Ig titers against purified NA ectodomain of A/California/04/09.

g Mice primed with PBS alone.

h Pooled convalescent sera from Balb/c mice infected with wild-type A/California/04/09.

### The neuraminidase of seasonal influenza viruses stimulate cross-reactive antibodies to purified pNA-ectodomain protein in mice

**Figure 2:**
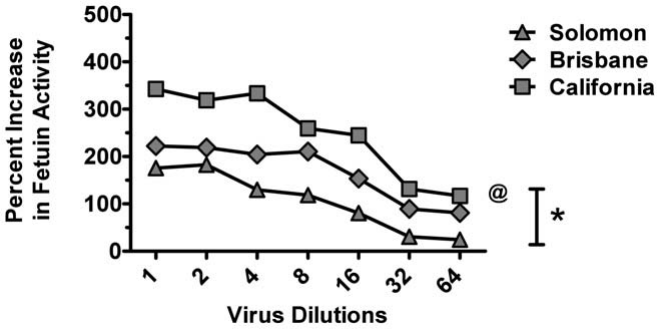
Enzymatic analysis of the NA activity between recombinant viruses. A panel of 7+1 rg recombinant viruses was generated by reverse genetics. Each recombinant virus contained the 7 gene segments from the PR8 strain and the NA gene from Solomon, Brisbane, or California. Using a 96-well plate, the activity of each NA in serial two fold dilutions of virus was determined by calculating the percent increase in cleavage of the substrate fetuin between each virus and PBS only wells. Activity was measured at an O.D. of 550 nm. Significant differences in mean activity were seen between rg viruses (*P = 0.007). The overall activity of wt California was higher than that of rg Solomon (@P<0.05) but not rg Brisbane (*P*>0.05). Data is representative of three independent experiments.

To address whether exposure to seasonal influenza strains could elicit cross-reactive antibodies to the NA of pH1N1, we generated several 7+1 rg viruses encoding the NA gene of Solomon, Brisbane, or California with 7 remaining PR8 genes. Because the only gene in these recombinant viruses that differed was the NA, we tested the NA activity of all viruses. Each virus preparation was serially diluted two fold and tested for NA activity by the miniaturized format of the NA assay as previously mentioned [20], [22]. Significant differences in mean activity (cleavage of fetuin substrate) were seen between recombinant viruses (Fig. 2) (*P = 0.007). The overall activity of wt California was higher than that of rg Solomon (@P<0.05) but not rg Brisbane (*P*>0.05).

Several wt seasonal influenza viruses induce cross-reactive serum antibodies to HA or NA of pH1N1 [10], [11], [20]. To establish whether the NA of seasonal H1N1 strains can stimulate cross-reactive antibodies to pH1N1 NA, Balb/c mice were infected with an equivalent dose (10^3^ EID_50_) of a single recombinant virus (rg Solomon, rg Brisbane, rg California). All groups were compared to mice inoculated with PBS or wt California as a negative or positive control, respectively. As predicted, each recombinant virus induced high and similar neutralizing serum antibody titers (HI) to the HA of PR8 (homologous) in mice (Table 1), suggesting that each virus stimulated an overall immune response of equivalent magnitude. In agreement with previous observations [14]–[16], [32], no HI titers were detected (<40) to the HA of wt California in animals infected with seasonal recombinant viruses. As predicted, sera collected from mice infected with the unrelated rg X−31(H3N2 A/Aichi/02/68) displayed similar HI titers to PR8 and no reactivity to the HA of wt California (Table 1). We next assessed cross-reactive antibodies using purified pNA-ecto by ELISA. Infection with rg Brisbane or rg Solomon induced 16–32-fold less pNA-ecto titers (320–640) than compared to rg California (10,240) as measured by ELISA. Nevertheless, pNA-ecto titers in animals infected with rg Solomon or rg Brisbane were markedly higher than titers in PBS-inoculated (40) or rg X−31 infected mice (80). As expected, no pH1N1-specific HI titers were detected in animals receiving PBS alone (<40) (Table 1). These results demonstrate that the antibodies to the NA of contemporary seasonal H1N1 strains have cross- reactivity to the NA of California.

### Antibodies to the neuraminidase of seasonal H1N1 viruses associate with immunity to pH1N1

**Figure 3:**
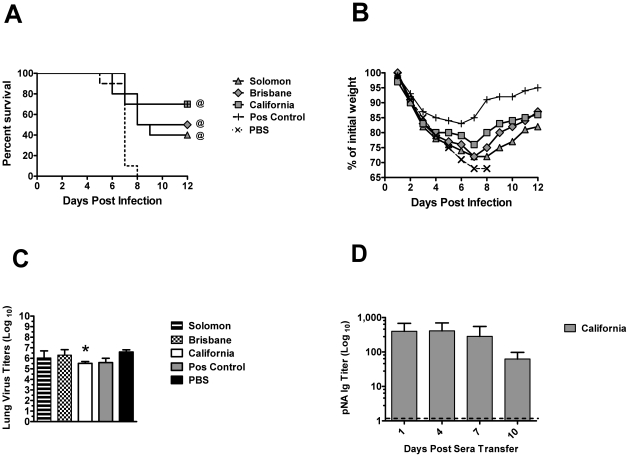
The effect of N1NA antibodies on immunity to pandemic H1N1 2009 challenge. Naïve Balb/c mice were injected intraperitoneally with pooled convalescent sera collected from mice infected with 7+1 rg recombinant viruses. Control mice were injected with either sera from mice inoculated with PBS or wt California. All passively transferred mice were challenged with a lethal dose (10^6^ EID_50_) of wt pH1N1 virus. Survival, weight loss and virus titers were monitored post challenge. A) Percent survival was measured between animals daily for 12 days post virus challenge; differences between rg California and PBS: *P*<0.001. Comparison between rg Solomon or rg Brisbane to PBS: *P* <0.02. B) Average weight loss in each treated group after virus challenge was monitored daily for 12 days. C) Virus titers in the lungs of all challenged mice at 5 dpi assessed by TCID_50_. The persistence of passively transferred antibodies in the blood stream was assessed using antibody-deficient SCID mice. Titers of cross-reactive antibodies to pNA-ecto were determined by ELISA. D) Differences in total Ig (IgM, IgA, IgG) titers were measured in the serum of SCID mice at 1 (*P* = 0.03), 4 (*P* = 0.03), 7 (*P* = 0.10) and 10 (*P* = 0.02) days post passive transfer with rg California. Data is representative of two independent experiments.

Mice immunized with DNA encoding the NA of the seasonal H1N1 strain, A/New Caledonia/20/99 were found to have reduced weight loss and enhanced survival upon lethal H5N1 challenge [18], demonstrating a role for NA in heterologous immunity. Because sera from mice inoculated with rg Solomon, rg Brisbane or rg California displayed alike HI titers but a 16–32 fold difference in anti-pNA-ecto antibody titers when compared to rg California sera (Table 1), we hypothesized that this would correlate with a reduction in immunity to pH1N1.To address this, sera from animals previously inoculated with recombinant viruses or either PBS (negative control) or wt California (positive control) were passively transferred into Balb/c mice. Recipient animals were then challenged with a lethal dose of wt California. Mice receiving rg California sera displayed increased survival when compared to animals receiving sera from negative control mice (*P*<0.001) (Fig. 3A). Also, rg Solomon or rg Brisbane sera protected better than that of PBS sera (*P*⩽0.02). Interestingly, transfer with rg California sera or positive control sera resulted in an equivalent percentage of survival in mice (70%) (*P* = 0.03). However, positive control sera provided the best protection from weight loss compared to all sera tested (Fig. 3B). Sera collected from mice infected with rg X−31 displayed equivalent percent survival to that of PBS sera (10%) (Fig. S3A). Mice transferred with either rg X−31 or PBS sera loss weight at similar kinetics for up to 6 dpi with the exception of one single mouse that recovered in each group (Fig. S3B). The low percent protection induced by rg X−31 sera was associated with minimal antibody reactivity to pNA (Table 1). Therefore, the N1 of contemporary seasonal influenza strains contributed greater cross-immunity than that of an unrelated older N2 strain. These data indicate that NA antibodies derived from different seasonal H1N1 strains afford alike cross-immunity against lethal pH1N1 infection. Also, the titer of cross-reactive antibodies to pNA-ecto associates with partial protection against lethality.

We then determined if cross-reactive NA antibodies were associated with a reduction in virus replication. Expectedly, virus titers in PBS recipient mice were greater than that of titers in mice receiving rg California sera at 5 dpi (*P*<0.05) (Fig. 3C). Although positive control sera protected 70% of mice from lethality, surprisingly, this sera did not significantly reduce virus replication in the lung when compared with PBS sera (*P*>0.05). No difference was observed between either rg Solomon or rg Brisbane and PBS sera (*P*>0.05). Because NA serum antibodies increased survival from lethal challenge, we tested how long passively acquired antibodies persisted in the blood stream several days post transfer. To test this, rg California sera were passively transferred into antibody-deficient SCID animals prior to wt pH1N1 challenge and pNA-ecto-specific antibodies were measured 1, 4, 7 and 10 days post transfer (Fig. 3D). Considerably high titers of antibodies were observed out to at least 10 dpi in SCID mice that received rg California sera. The persistence of these antibodies however did not diminish virus replication in the lung of any SCID animal when compared to PBS-inoculated SCID mice (*P*≥0.45) (data not shown) demonstrating that these passively-acquired antibodies were not sufficient for virus clearance.

### Multiple exposures to wild-type seasonal viruses provide cross-reactive immune responses to the neuraminidase of pandemic influenza virus

Humans are likely to be exposed to different strains of seasonal influenza viruses throughout their lifetime. In ferrets, the magnitude of the cross-protective response to pH1N1 is enhanced if animals are infected twice with wt seasonal influenza viruses [16]. It has been shown previously that the immune response to NA is impaired by previous exposure to related HA [33], [34]. To determine whether multiple exposures to contemporary wt seasonal strains can contribute to cross-reactive antibodies to the NA of pH1N1, Balb/c mice were primed with wt Solomon or wt Brisbane then three weeks later boosted with wt Solomon. As expected, priming with wt Solomon induced high HI titers post prime (1,280) and boost (1,280) against homologous virus (Table 2). The absence of increased homologous titers post boost suggested that these mice were at least marginally protected from secondary homologous infection. Mice that were primed with wt Brisbane displayed an eight fold less HI titer (160) to wt Solomon than homologous boost. As seen in previous studies [14]–[16], [32], we could not detect HI titers to wt California after exposure to contemporary seasonal influenza strains. Expectedly, we saw a considerably high level of response to the pNA-ecto of wt California in mice primed with wt Tennessee (1,440–4,022). There was equivalent cross-reactive pNA-ecto titers post boost in mice primed with wt Brisbane (320) or wt Solomon (320). However, boosting with wt Solomon reproducibly increased the cross-reactive titers to pNA-ecto in mice primed with either wt Solomon or wt Brisbane (160 to 320). In separate studies, priming with swine-origin H1N1 A/New Jersey/11/76 (wt New Jersey) enhanced the cross-reactive pNA-ecto titers after boost with wt Solomon (1280) (data not shown). This is in agreement with the observation that previous exposure to wt New Jersey boosts pre-existing cross-reactive antibodies to pH1N1 in elderly humans [10]. These individuals likely underwent natural infection with contemporary seasonal viruses. As predicted, animals inoculated with only the wt Tennessee developed a strong reactivity to pNA-ecto (102,400–204,800). Therefore, our results strongly mirror the scenario of multiple exposures in nature and suggest that cross-reactive pNA-ecto titers induced by repeated exposure to seasonal influenza strains may contribute to reducing the severity of pH1N1 infection.

**Table 2:** Serum antibody titers in Balb/c mice after prime or boost with wild-type viruses as determined by hemagglutination inhibition and ELISA assays.

GROUP a	HI	HI	ELISA (Ig) b
Solomon/ Solomon	Solomon HA c	California HA d	California NA e
Post Prime	1,280	<40	160
Post Boost	1,280	<40	320
**Brisbane/ Solomon**			
Post Prime	<40	<40	160
Post Boost	160	<40	320
**Tennessee/ PBS** f			
Post Prime	<40	1,440	204,800
Post Boost	<40	4,022	102,400

a Each group of mice primed (0 dpi) and boosted (21 dpi) intranasally with wild-type H1N1 influenza viruses.

b ELISA titers expressed as reciprocal of the highest dilution of pooled sera with an O.D. value of≥0.150 when tested against purified NA ectodomain of A/California/04/09.

c Pooled sera collected three weeks post prime or boost and tested for HI titers against wild-type A/Solomon/03/06.

d Pooled sera collected three weeks post prime or boost and tested for HI titers against wild-type A/California/04/09.

e Pooled sera tested for Ig titers against purified NA ectodomain of A/California/04/09.

f Mice primed with A/Tennessee/1-560/09 and boosted with PBS alone.

## Discussion

The goal of the current study was to define two parameters: [i] The extent of cross reactivity of antibodies raised against the NA of antigenically distinct [10], [12], [31] seasonal H1N1 influenza strains to the NA of pH1N1 and [ii] The contribution of these cross-reactive antibodies on the outcome of severe wt pH1N1 infection. Here we show that exposure to the NA of contemporary H1N1 seasonal strains induce antibodies with high reactivity to pNA-ecto. Furthermore, these antibodies associate with immunity albeit marginally against pH1N1-related lethality. Using wt seasonal viruses, we also show that multiple exposures with these wt strains resulted in reactivity to pNA-ecto. These studies demonstrate that the HA is not the sole immune correlate in protection from a severe pH1N1infection, but in addition to HA, the NA of seasonal influenza plays a contributing role in inducing cross reactive-immune responses to antigenically variant strains. The magnitude of cross-reactive HA antibodies in response to pH1N1 has been described elsewhere [10]. Several limitations exist when protective responses are determined by antibody responses to HA alone. Measuring exclusively HA antibodies may severely underestimate the degree of immunity by omitting the detection of antibodies raised against other viral proteins [35]. In our previous study [20], we discovered that antibodies to NA did not positively correlate with antibodies to HA, thus supporting this idea.

We cannot rule out that inadequate protection mediated by seasonal NA antibodies was due to the differences in NA content, NA activity of recombinant viruses, antigenic inhibition by PR8 HA or slight differences in virus replication between the recombinant viruses. The generation of purified pNA-ecto provided a useful tool to also address antigenicity by an ELISA-based approach. In theory, the NA content of rg California viruses may be addressed using the homologous pNA-ecto protein, however, a method to calculate the precise concentration of NA in each virus preparation is currently lacking. Regrettably, our inability to normalize the NA concentration in each recombinant virus tested presented a limitation in our studies. However, mice infected with these recombinant viruses responded with equivalent HI titers to homologous (PR8) and heterologous (pHA) proteins. Novel approaches of determining NA concentrations in virus preparations are necessary to begin to address the effect of NA content on antibody-based protection. Nevertheless, some data suggest that the NA activity of influenza vaccine preparations may be used as a marker for NA content and standardization [21]. Previously, it has been show that the activity of NA is correlated with the magnitude of the antibody response to this protein [21], [36], [37]. In our current studies, we observed a marked difference in NA activity between rg Solomon versus rg California. We do not believe that the cross-reactive antibody response to pNA-ecto was affected by NA activity. This is supported by the observation that infection with a rg PR8 virus containing the NA of PR8 induced 4–8 fold less cross-reactive antibodies in mice but retained significantly higher NA activity than that of rg Solomon (*P*<0.05) and rg Brisbane (*P*<0.05) NA proteins (data not shown). It is unlikely that insufficient antibody-mediated immunity to pH1N1 was partially due to antigenic competition by PR8 HA because we previously demonstrated that the HA of PR8 does not alter recognition to or activity of NA [20]. Although not directly tested in our studies, the kinetics of virus replication between the rg viruses may have differed, thus altering the immune responses in mice. Assuming this to be true, the fact that all passively transferred sera were normalized in their HI titer, yet varied in the level of anti-pNA-ecto antibodies support our hypothesis that NA antibodies contributed to cross-immunity.

Because of the presence of other virus proteins (e.g, NP, HA), we acknowledge that in our current studies, the full degree of immunity mediated by anti-NA antibodies was not established. Since we utilized live viruses in our studies rather than single NA constructs, we could not preclude a contributing role for various PR8 proteins. We concede that the presence of non NA proteins of PR8 may have added to immunity against pH1N1. In our report, the NA was presented in the context of an influenza infection and therefore studied as an indirect measure of the role NA in immunity to pH1N1. The HI titers (homologous and heterologous) in sera induced by the recombinant viruses were alike, while anti-pNA-ecto antibodies varied, suggesting that NA antibodies contribute to protection. This was confirmed in a separate preliminary study where convalescent sera from wt pH1N1 infection was absorbed to pNA-ecto and therefore, partially depleted of NA antibodies. The partially-depleted sera afforded less protection (than non-depleted sera) from pH1N1 challenge when passively transferred into naïve Balb/c mice (data not shown). We conclude that antibodies to NA contributed but were not the sole antibody that afforded immunity against pH1N1 challenge.

We used live viruses to mirror the scenario of multiple infections where humans develop varying levels of cross-reactive responses throughout lifetime, which is largely dependent on the number of exposures. With repeated exposures, humans are presumably more equipped immunologically to deal with pH1N1 infection. Our findings using wt seasonal strains are in agreement with previous results that demonstrate repeated infections or vaccination with seasonal strains [16], [38] is capable of inducing cross-reactive responses to pH1N1. It has been shown that the immune response to NA is impaired by competition by previous exposure to closely-associated HA [39], [40]. It was unlikely that responses to NA were inhibited by previous exposure to homologous HA in our studies using wt Solomon since we observed an increase in anti-pNA-ecto titer after boost. Our results support previous studies using DNA or purified NA constructs, which show a contributing role for anti-NA antibodies in protection against heterologous virus challenge [1], [18], [41].

The mechanism by which cross-reactive antibodies to seasonal NA may contribute to protection against pH1N1 is unknown. However, the well-established role for NA antibodies is to provide a reduction in virus replication [42], [43], or cross-linking of virus particles on infected cells [44] or enhancing complement-mediated pathways [45] which can all likely diminish disease severity [19], [46]. In our studies however, we saw a trend of reduced virus replication in the lungs using sera from mice transferred with rg California or positive control antibodies. However, the difference reached statistical significance only when rg California, not positive control sera were administered. We cannot account for this unexpected result. This may be due to the high lethal dose of the virus used to challenge mice. Future studies are necessary to determine whether sera can diminish virus replication using sub-lethal doses of pH1N1. Despite the lack of reduction of virus replication using serum antibodies induced by seasonal recombinant viruses, we found that mice passively transferred seasonal NA-specific antibodies survived lethal pH1N1infection longer than mice transferred naïve sera (PBS). This suggests that a reduction of virus replication is not the sole mechanism of anti-NA antibody-mediated immunity in our model. Studies to identify the method employed by these cross-reactive serum antibodies are required to address the mechanism of cross-immunity.

It is more likely that cross-immunity generated by exposure to seasonal H1N1 influenza strains require the coordinated efforts of not merely HA and NA antibodies but also cross-reactive memory T lymphocytes responses [47]–[49]. This is supported by the observation that in our studies, SCID mice lacking B and T lymphocytes developed uncontrolled virus replication in the lungs after transfer with rg sera (data not shown). The substantial conservation of several non-NA major histo-compatibility complex-1 epitopes between seasonal influenza strains and that of pH1N1 support a role for T lymphocytes in cross-reactive immunity [30]. Studies in mice where two inoculations with wt Solomon or wt Brisbane and in separate studies, wt New Jersey (data not shown), show a strong cross-reactive antibody response to pNA-ecto which was likely accompanied by substantial cross-reactive CD4^+^ and CD8^+^ T lymphocyte responses. An important function of cross-reactive T lymphocytes is the secretion of cytokines [47], [50]. Cytokines play an important role in protection against influenza viruses [51]–[53]. We saw a 9.6 fold increase in only IL-15 in rg X−31 versus PBS sera (data not shown). However, it is unlikely that the presence of this cytokine impacted immunity upon adoptive transfer, because rg-X–31 sera afforded merely 10% survival from challenge. These results demonstrate that the partial immunity observed after passive transfer was due to the presence of antibody.

Our results are in agreement with findings that reveal an important role for exposure to seasonal H1N1 influenza viruses via natural infection or vaccination in the generation of cross-reactive immune responses to pH1N1 [4], [10], [11], [16], [17], [48], [49], [54], [55]. However, we address for the first time the contributing role of serum N1 NA antibodies in the cross-protection against pH1N1-related severity. Our results mirror findings that show N2 fail to confer cross-immunity against the genetically distinct H1N1 subtype [19]. In light of our recent findings, we propose that in addition to the HA protein, the NA content must be measured and optimized in future influenza vaccine formulations. Furthermore, our findings agree with the observation that in some cases, antibodies to HA are not always a surrogate marker for immunity [36]. A report indicated that the supplementation of NA from seasonal strains into conventional seasonal vaccines may help to broaden the immune response [56]. Therefore, the immune response to the NA protein will be an important factor to consider in the planning for future pandemics or the possible re-emergence of a more virulent pH1N1 strain.

## Supporting Information

Figure S1 **Analysis of recombinant NA protein of pandemic H1N1 2009 virus.** The NA ectodomain protein of the A/California/04/09 strain (pNA-ecto) was expressed using a baculovirus insect cell expression system and purified (materials and methods). A**)** Size-exclusion chromatography of recombinant NA protein. Purified pNA-ecto protein elutes as a well-folded tetramer (labeled black peak) with an apparent molecular size of∼200 kDa compared to molecular size standards (labeled gray peaks) on a Superdex 200 size-exclusion chromatography column. B) Denaturing Coomassie-stained gel of purified tetrameric pNA-ecto (*) compared to molecular weight standards (kDa).
(TIF)

Figure S2 **Activity and specific reactivity of purified pNA-ecto protein.** The activity of serial two-fold dilutions of purified pNA-ecto in the presence or absence of CaCl_2_ was tested. A) Activity was determined by calculating the percent increase in cleavage of the substrate fetuin between pNA-ecto and PBS only wells. Activity was measured at O.D. of 550 nm. B) ELISA were performed using sera collected from humans (65–93 yrs old) 4 weeks post immunization with TIV containing either Solomon or Brisbane H1N1 components. Pooled sera from mice inoculated with PBS or infected wt California were used as negative, positive controls respectively. Purified pNA-ecto was used to test the specific reactivity of human and animal sera in all assays. Individuals immunized with either TIV Solomon or Brisbane H1N1 developed a large degree of cross-reactive Ig antibodies to pNA-ecto by ELISA. Little reactivity was observed in negative control sera. Conversely, high levels of Ig titers were seen in mice infected with homologous wt virus. Observed differences in Ig titers detected between Solomon and Brisbane human sera (*P*≥0.27). Data is representative of two independent experiments.
(TIF)

Figure S3 **Analysis of anti N2 antibodies on protection against pandemic H1N1 2009 virus.** Naïve Balb/c mice were injected intraperitoneally with pooled sera collected from mice infected with 7+1 rg X−31 or inoculated with PBS. All passively transferred mice were challenged with a lethal dose (10^6^ EID_50_) of wt pH1N1 virus. Survival and weight loss were monitored post challenge. A) Percent survival was measured between animals daily for 12 days post virus challenge. B) Average weight loss in each treated group after virus challenge was monitored daily for 12 days. Data is representative of two independent experiments.
(TIF)
