# Supplementary figures and images for: A Contributing Role for Anti-Neuraminidase Antibodies on Immunity to Pandemic H1N1 2009 Influenza A Virus
Source: PLoS One. 2011 Oct 24;6(10):e26335. doi: 10.1371/journal.pone.0026335 (PMC3200314; doi:10.1371/journal.pone.0026335)

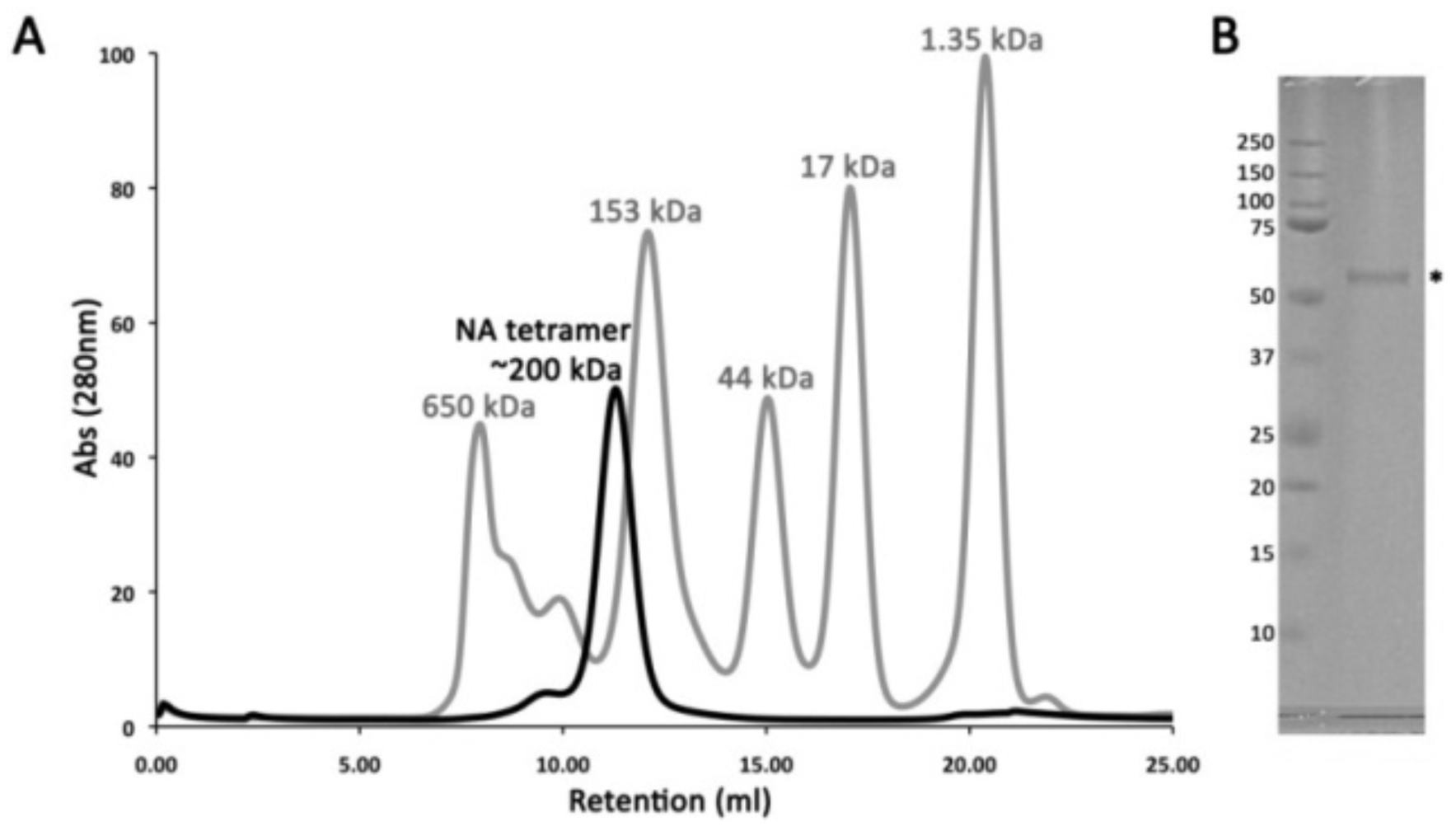

Supplement: Figure S1 — Analysis of recombinant NA protein of pandemic H1N1 2009 virus. The NA ectodomain protein of the A/California/04/09 strain (pNA-ecto) was expressed using a baculovirus insect cell expression system and purified (materials and methods). A) Size-exclusion chromatography of recombinant NA protein. Purified pNA-ecto protein elutes as a well-folded tetramer (labeled black peak) with an apparent molecular size of∼200 kDa compared to molecular size standards (labeled gray peaks) on a Superdex 200 size-exclusion chromatography column. B) Denaturing Coomassie-stained gel of purified tetrameric pNA-ecto (*) compared to molecular weight standards (kDa). (TIF) [file pone.0026335.s001.tif]

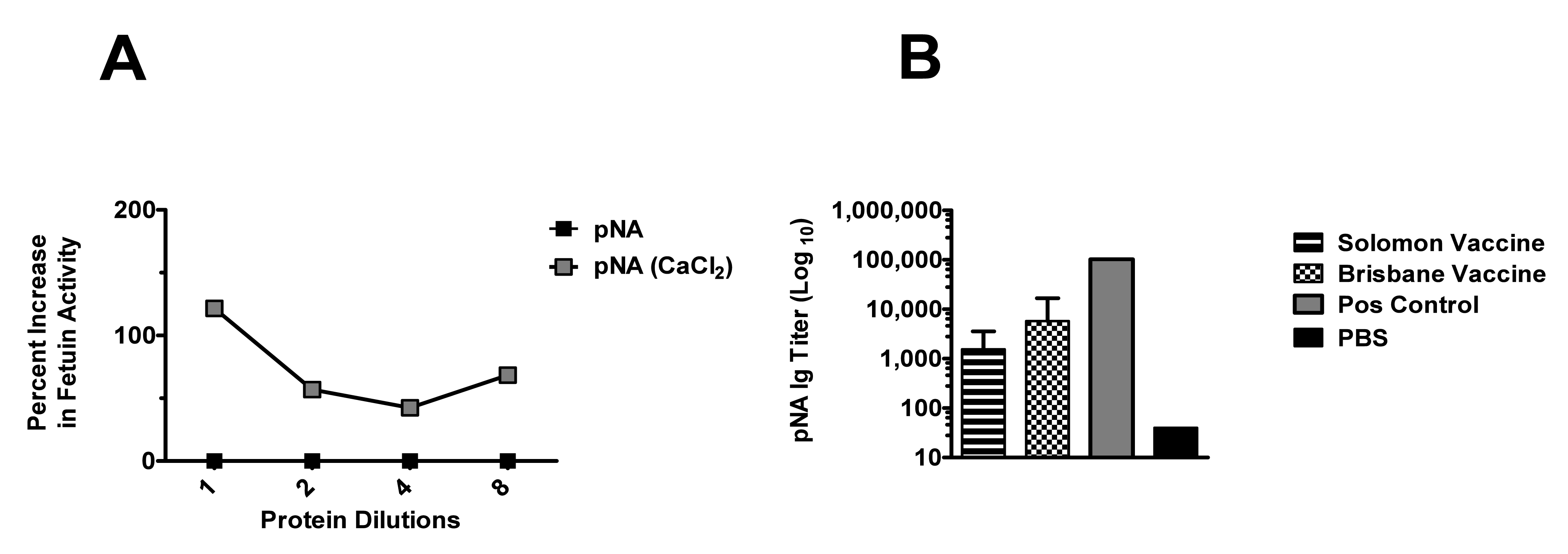

Supplement: Figure S2 — Activity and specific reactivity of purified pNA-ecto protein. The activity of serial two-fold dilutions of purified pNA-ecto in the presence or absence of CaCl2 was tested. A) Activity was determined by calculating the percent increase in cleavage of the substrate fetuin between pNA-ecto and PBS only wells. Activity was measured at O.D. of 550 nm. B) ELISA were performed using sera collected from humans (65–93 yrs old) 4 weeks post immunization with TIV containing either Solomon or Brisbane H1N1 components. Pooled sera from mice inoculated with PBS or infected wt California were used as negative, positive controls respectively. Purified pNA-ecto was used to test the specific reactivity of human and animal sera in all assays. Individuals immunized with either TIV Solomon or Brisbane H1N1 developed a large degree of cross-reactive Ig antibodies to pNA-ecto by ELISA. Little reactivity was observed in negative control sera. Conversely, high levels of Ig titers were seen in mice infected with homologous wt virus. Observed differences in Ig titers detected between Solomon and Brisbane human sera (P≥0.27). Data is representative of two independent experiments. (TIF) [file pone.0026335.s002.tif]

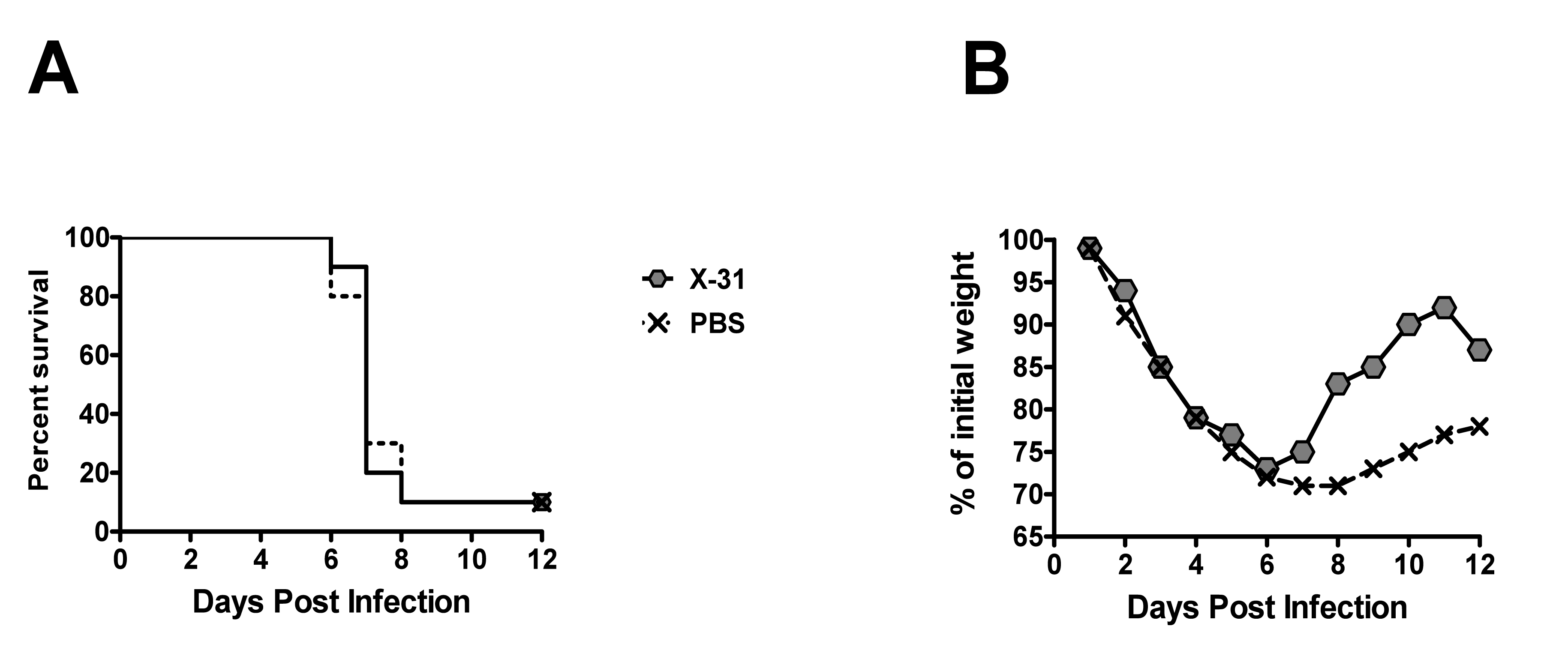

Supplement: Figure S3 — Analysis of anti N2 antibodies on protection against pandemic H1N1 2009 virus. Naïve Balb/c mice were injected intraperitoneally with pooled sera collected from mice infected with 7+1 rg X−31 or inoculated with PBS. All passively transferred mice were challenged with a lethal dose (106 EID50) of wt pH1N1 virus. Survival and weight loss were monitored post challenge. A) Percent survival was measured between animals daily for 12 days post virus challenge. B) Average weight loss in each treated group after virus challenge was monitored daily for 12 days. Data is representative of two independent experiments. (TIF) [file pone.0026335.s003.tif]
